# Supplementary material for: Set the tone: Trustworthy and dominant novel voices classification using explicit judgement and machine learning techniques
Source: PLoS One. 2022 Jun 29;17(6):e0267432. doi: 10.1371/journal.pone.0267432 (PMC9242519; doi:10.1371/journal.pone.0267432)
Supplement: S1 File — (DOCX) [file pone.0267432.s001.docx]

# **Supporting information**

**S1 Text. Sentences provided to the speaker in the study’s language (french).**

Original sentences used in the study were (in french) « Je pense être le candidat adapté pour ce poste » (translation: « I think I’m a worthy candidate for this position »), « Je remplis les qualifications nécessaires pour effectuer cette mission » (translation: « I am qualified to carry out this task »), « J’ai les compérences requises pour mener à bien cette mission » (translation: « I have the required competences to carry out this task ») and «Je pense avoir les compétences nécessaires pour ce poste » (translation: «I think I have required competencies for that position»).

**S2 Text.** **Summary of the acoustic measurements.**

Measures related to fundamental frequency comprised cues of mean fundamental frequency (Hz, primary voice harmonic), minimum fundamental frequency (Hz), maximum fundamental frequency (Hz), standard deviation of f0 (Hz) which provides indications of f0’s medium term instability, intonation (Hz; measured by subtracting minimum f0 to maximum f0, (McAleer et al., 2014)), first to fifth quantiles of f0 (0-0.05Hz to 0.95; as described in Praat Manual, [66])Secondly, intensity-related measures comprised mean intensity (MeanIntensity, [dB]), minimum intensity (MinIntensity,[dB]), maximum intensity (MaxIntensity, [dB]), standard deviation of intensity (SdIntensity, [dB]) which can be understood as intensity’s medium term instability. Speech rate is the only time-related measure that was analysed. It was extracted using De Jong and Wempe’s open-source script [68] and calculates the number of syllables per second. Following Banse and Scherer’s [69] methodology, complex measures of voice quality related to spectral balance were estimated by means of the distribution of energy in the low-frequency range (PE500: Energy below 500Hz) and the high frequency range (PE1000: Energy below 1000Hz), the difference between maximum energy in the 0-2kHz and 2-5kHz ranges (i.e. the Hammarberg index [dB], [70]), amplitude difference between the first and second harmonics (h1-h2 [dB]), cepstral peak prominence (CPP), spectral center of gravity (COG [Hz]), formant dispersion up to the third formant (*Df* F1-F3, [Hz])), and the fifth formant (*Df* F1-F5, [Hz]). Voice quality parameters related to variability were measured by means of i) Jitter, a mean absolute difference between consecutive intervals (glottal period), divided by the average interval, ii) Shimmers, a measure of amplitude variability between consecutive intervals, divided by the average interval, iii) Harmonics-to-noise ratin (HNR, [dB]), measuring the degree of acoustic periodicity, iv) additionally, distribution of energy over the spectrum at fifteen overlapping frequency bands of 500Hz bandwidths (from 0 to 4000Hz, with 250Hz increments) was measured and referred to by their medium value (Ex. “Energy250” refers to energy between 0 and 500Hz) [71].


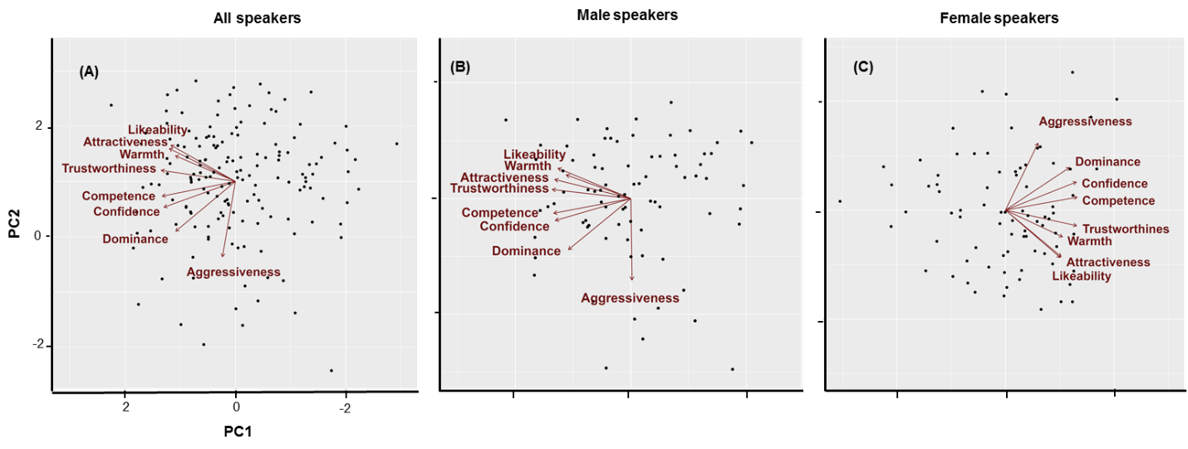


**Supplementary Figure S1. 2D-space representation of principal component analysis of attitude judgment from voices.** Each voice stimulus is represented on a bipolar scale of two components: PC1 (which can be interpreted as a valence evaluation, where Likeability saturates with the highest score) on X-axis and PC2 (which can be interpreted as dominance evaluation, where Aggressiveness saturates with the highest score) on the Y-axis.

S1 Table. Reliability of judgments, measured by Cronbach alpha scores, by scale

| Scale | Cronbach’s Alpha |
| --- | --- |
| likability | 0.81 |
| aggressiveness | 0.83 |
| attractiveness | 0.81 |
| warmth | 0.81 |
| competence | 0.80 |
| trustworthiness | 0.80 |
| dominance | 0.80 |
| feminity | 0.90 |
| masculinity | 0.89 |
| confidence | 0.79 |

S2 Table. Evaluation results of the vocal profiles (mean, SD, N)

| Scale | Mean | Std. Deviation | N |
| --- | --- | --- | --- |
| likability | 43.02 | 16.942 | 168 |
| aggressiveness | 24.67 | 13.948 | 168 |
| attractiveness | 40.02 | 17.463 | 168 |
| warmth | 41.48 | 17.126 | 168 |
| competence | 45.85 | 16.743 | 168 |
| trustworthiness | 43.73 | 17.061 | 168 |
| dominance | 39.29 | 19.515 | 168 |
| feminity | 40.57 | 32.479 | 168 |
| masculinity | 40.16 | 31.997 | 168 |
| confidence | 46.74 | 19.784 | 168 |

S3 Table. Coefficients summary of stepwise regression analyses for acoustical parameters and perceived dimensions, by sex.

|  | Sex | Model | Unstandardized Coefficients | | Standardized Coefficients | *t* | *p* | 95.0% Confidence Interval for B | |
| --- | --- | --- | --- | --- | --- | --- | --- | --- | --- |
| Dimension |  |  | *B* | Std. Error | β |  |  | Lower Bound | Upper Bound |
| Valence | all | (Constant) | -2.23 | 0.59 |  | -3.79 | 0.00 | -3.39 | -1.07 |
|  |  | Center of gravity | 0.00 | 0.00 | 0.20 | 2.56 | 0.01 | 0.00 | 0.00 |
|  |  | Shimmer | 9.61 | 2.78 | 0.27 | 3.46 | 0.00 | 4.13 | 15.10 |
|  |  | Intensity min. | 0.03 | 0.01 | 0.17 | 2.11 | 0.04 | 0.00 | 0.05 |
|  | f | (Constant) | -1.57 | 0.46 |  | -3.41 | 0.00 | -2.48 | -0.65 |
|  |  | Shimmer | 16.34 | 4.66 | 0.37 | 3.51 | 0.00 | 7.06 | 25.62 |
|  | m | (Constant) | 6.14 | 2.02 |  | 3.04 | 0.00 | 2.12 | 10.17 |
|  |  | Quantile 1 | -0.08 | 0.02 | -0.93 | -4.72 | 0.00 | -0.12 | -0.05 |
|  |  | PE1000 | -2.92 | 1.74 | -0.19 | -1.68 | 0.10 | -6.39 | 0.55 |
|  |  | F0 min. | 0.04 | 0.01 | 0.47 | 2.66 | 0.01 | 0.01 | 0.06 |
|  |  | Quantile 5 | 0.01 | 0.01 | 0.28 | 2.13 | 0.04 | 0.00 | 0.02 |
| Dominance | all | (Constant) | 1.75 | 0.37 |  | 4.71 | 0.00 | 1.01 | 2.48 |
|  |  | Energy3250 | 0.09 | 0.01 | 0.49 | 7.06 | 0.00 | 0.07 | 0.12 |
|  |  | HNR | -0.14 | 0.03 | -0.31 | -4.05 | 0.00 | -0.21 | -0.07 |
|  |  | Quantile 4 | 0.00 | 0.00 | 0.27 | 3.38 | 0.00 | 0.00 | 0.01 |
|  |  | *Df* (F1-F5) | 0.00 | 0.00 | -0.14 | -1.99 | 0.05 | 0.00 | 0.00 |
|  | f | (Constant) | 0.35 | 1.03 |  | 0.33 | 0.74 | -1.71 | 2.40 |
|  |  | HNR | -0.21 | 0.04 | -0.44 | -4.92 | 0.00 | -0.29 | -0.12 |
|  |  | Energy250 | 0.11 | 0.03 | 0.53 | 4.24 | 0.00 | 0.06 | 0.16 |
|  |  | *Df* (F1-F3) | 0.00 | 0.00 | 0.17 | 1.97 | 0.05 | 0.00 | 0.00 |
|  |  | Speech rate | 0.32 | 0.11 | 0.24 | 2.84 | 0.01 | 0.09 | 0.54 |
|  |  | Energy3250 | 0.08 | 0.02 | 0.34 | 3.56 | 0.00 | 0.04 | 0.13 |
|  |  | Energy500 | -0.12 | 0.04 | -0.42 | -3.18 | 0.00 | -0.20 | -0.05 |
|  | m | (Constant) | 2.91 | 0.66 |  | 4.40 | 0.00 | 1.59 | 4.22 |
|  |  | Energy3250 | 0.19 | 0.05 | 1.19 | 3.63 | 0.00 | 0.09 | 0.30 |
|  |  | PE500 | -2.57 | 0.79 | -0.30 | -3.25 | 0.00 | -4.15 | -1.00 |
|  |  | Energy3500 | -0.12 | 0.06 | -0.69 | -2.07 | 0.04 | -0.23 | 0.00 |

*Note.* The table represents the summary of six independent stepwise regression analyses, for dimension by sex groups (2x3), when sex is either considered as a splitting variable (females [f], males [m]), or weld together (all). For each analysis, all displayed predictors are entered to the model and kept after reaching a probability of F-to-enter <= 0.05, and a probability of F-to-remove >=.10. Excluded variables are not displayed, for visibility.

S4 Table. Summary of selected features for SVM and RF classification amongst all measured acoustics, based on their relative weight

|  |  | SVM classifier | | | | RF |  |
| --- | --- | --- | --- | --- | --- | --- | --- |
|  |  | 2D | Dominance | Valence | 2D | | |
| Description | Parameter | accuracy: 77.78% | accuracy: 58.6% | accuracy: 52.1% | OOB: 34.92% | | |
| F0 parameters | F0 Mean | * |  |  |  | | |
|  | F0 Min. |  |  | * |  | | |
|  | F0 Max. |  | * | * |  | | |
|  | F0 Median | * |  |  |  | | |
|  | SD-F0 |  | * | * |  | | |
|  | Quantile 1 |  | * | * |  | | |
|  | Quantile 2 |  |  |  |  | | |
|  | Quantile 3 | * |  |  |  | | |
|  | Quantile 4 | * |  |  |  | | |
|  | Quantile 5 |  | * |  |  | | |
| Intensity parameters | Mean Intensity |  | * |  |  | | |
|  | Min. Intensity |  | * |  | * | | |
|  | Max. Intensity |  |  |  |  | | |
|  | Median Intensity |  |  |  |  | | |
|  | SD Intensity | * |  |  | * | | |
| Time related | Speech rate |  | * |  |  | | |
| Voice quality (spectral balance) | Energy below 500 Hz (PE500) |  |  |  |  | | |
|  | Energy below 1000Hz (PE1000) | * |  |  |  | | |
|  | Hammarberg index | * |  |  |  | | |
|  | H1-H2 | * |  |  |  | | |
|  | Cesptral Peak Prominence (CPP) | * | * |  |  | | |
|  | center of gravity (COG) |  |  |  |  | | |
|  | Formant dispersion D*f*(F1F3) |  |  | * |  | | |
|  | Formant dispersion D*f*(F1F5) |  | * |  |  | | |
| Voice quality (spectral energy distribution) | Energy250 |  |  |  |  | | |
|  | Energy500 |  |  |  | * | | |
|  | Energy750 | * |  |  |  | | |
|  | Energy1000 |  |  |  | * | | |
|  | Energy1250 |  |  |  |  | | |
|  | Energy1500 |  | * |  |  | | |
|  | Energy1750 |  |  | * | * | | |
|  | Energy2000 |  |  |  | * | | |
|  | Energy2250 |  | * |  | * | | |
|  | Energy2500 |  |  |  |  | | |
|  | Energy2750 |  |  |  |  | | |
|  | Energy3000 |  | * |  |  | | |
|  | Energy3250 | * |  |  | * | | |
|  | Energy3500 |  | * | * | * | | |
|  | Energy3750 |  |  | * |  | | |
| Voice quality (variability) | HNR |  | * |  | * | | |
|  | Jitter |  | * |  |  | | |
|  | Shimmer | * | * |  | * | | |

*Note*. (*) indicate a feature selected for the analysis.

S5 Table. Selected features for each classification method of bi-dimensional vocal profiles, based on relative feature importance

| Support Vector Machine | | Random Forest | |
| --- | --- | --- | --- |
| COG | MinIntensity | |  |
| PE1000 | Energy500 | |  |
| Quantile4 | HNR | |  |
| Hammarberg | Energy2500 | |  |
| CPP | **SDIntensity** | |  |
| **SDIntensity** | Energy1750 | |  |
| Medianf0 | Energy2250 | |  |
| **Energy3250** | Energy2000 | |  |
| Meanf0 | **Energy3250** | |  |
| Energy750 | Energy1000 | |  |
| Quantile3 | **Shimmer** | |  |
| H1H2 | Energy3500 | |  |
| **Shimmer** |  | |  |

*Note*. Features are reported by order of importance. Relevant features in both classification methods are reported in bold.

S6 Table. Selected features for each SVM classifier (bi-dimensional, power-dominance, valence-trustworthiness) based on relative feature importance

| 2D | Dominance | Valence |
| --- | --- | --- |
| COG | SDf0 ** | F0min |
| PE1000 | CPP ** | Df(F1F3) |
| Quantile4 | Energy3000 | Energy1750 |
| Hammarberg | Speech rate | F0max ** |
| CPP ** | MeanIntensity | Quantile1 ** |
| SDIntensity | Energy2250 | Energy3750 |
| Medianf0 | Shimmer ** | SDf0 ** |
| Energy3250 | Df(F1F5) | Energy3500 ** |
| Meanf0 | MinIntensity ** |  |
| Energy750 | Quantile1 ** |  |
| Quantile3 | Jitter |  |
| H1H2 | Energy1500 |  |
| Shimmer ** | HNR ** |  |
|  | F0max ** |  |
|  | Energy3500 ** |  |
|  | Quantile5 ** |  |

*Note.* Features are reported by order of importance. Relevant features in both classification methods are reported in bold.

** features selected in two classifiers


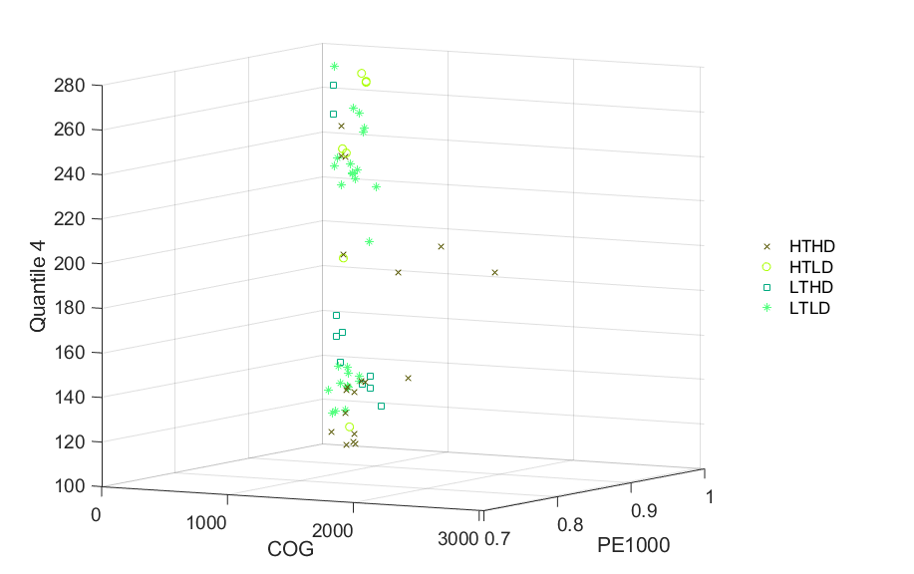


Supplementary Figure S2. Interaction of the three best features based on their NCA weight, relative to vocal categories (Y: Quantile4; X: COG; Z: PE1000), for the bi-dimensional classification.


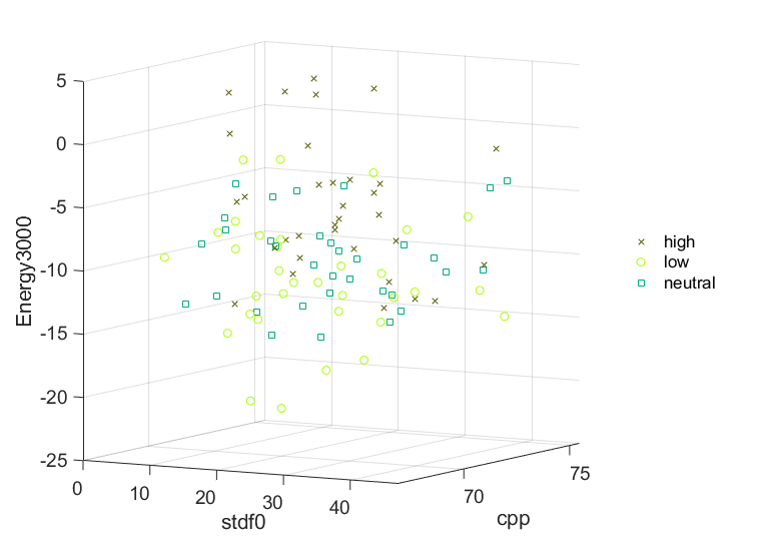


Supplementary Figure S3. Three-dimensional scatterplot based on the selected three best features after NCA (Y: Energy3000; X: SDf0; Z: CPP), for dominance classification.


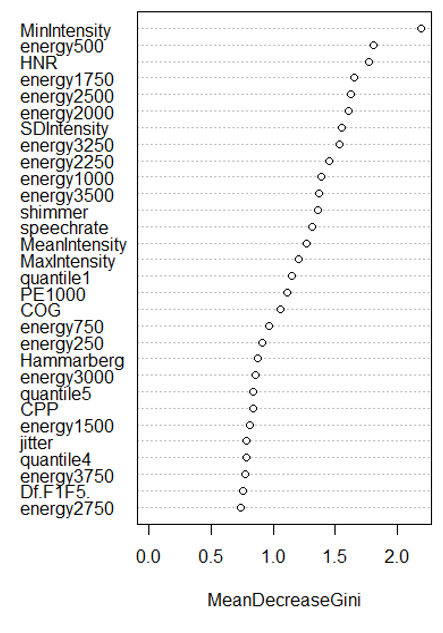


**Supplementary Figure S4.** **Acoustical features ordered by importance measure (mean decrease Gini), in the random forest analysis.**
