# Supplementary material for: Set the tone: Trustworthy and dominant novel voices classification using explicit judgement and machine learning techniques
Source: PLoS One. 2022 Jun 29;17(6):e0267432. doi: 10.1371/journal.pone.0267432 (PMC9242519; doi:10.1371/journal.pone.0267432)
Supplement: Attachment — Submitted filename: Response_review.docx [file pone.0267432.s002.docx]

**Manuscript PONE-D-21-09962**

**Response to Reviewers**

Dear Thippa Reddy Gadekallu,

Thank you for giving us the opportunity to submit a revised draft of the manuscript “Set the tone: Trustworthy and dominant novel voices classification using explicit judgement and machine learning techniques” (initially: “Set the tone: first impressions from voices and affective classification using supervised machine learning techniques”) for publication in the Journal Plos One. We appreciate the time and effort that you and the reviewers dedicated to providing feedback on our manuscript and are grateful for the insightful comments on and valuable improvements to our paper. We have incorporated most of the suggestions made by the reviewers. Those changes are highlighted within the manuscript using Microsoft Word’s Track Changes tool. Please see below, in blue, for a point-by-point response to the reviewers’ comments and concerns. All page numbers refer to the revised manuscript file with tracked changes.

## **Journal requirements:**

1. Please ensure that your manuscript meets PLOS ONE's style requirements, including those for file naming. The PLOS ONE style templates can be found at <https://journals.plos.org/plosone/s/file?id=wjVg/PLOSOne_formatting_sample_main_body.pdf> and <https://journals.plos.org/plosone/s/file?id=ba62/PLOSOne_formatting_sample_title_authors_affiliations.pdf>

This point has been modified accordingly.

2. Please consider changing the title so as to meet our title format requirement (<https://journals.plos.org/plosone/s/submission-guidelines>). In particular, the title should be "Specific, descriptive, concise, and comprehensible to readers outside the field" and in this case it is not informative and specific about your study's scope and methodology.

The title has been modified as to fit the requirements, and is now: “Set the tone: Trustworthy and dominant novel voices classification using explicit judgement and machine learning techniques”

3. Please improve statistical reporting and refer to p-values as "p<.001" instead of "p=.000". Our statistical reporting guidelines are available at <https://journals.plos.org/plosone/s/submission-guidelines#loc-statistical-reporting>.

Careful attention was given to this point: we screened the document and found a p-value referred to as an equality page 21, line 469. However, it describes a non-significant result, therefore we suggest to keep the actual P value in accordance with the statistical reporting guidelines (<https://journals.plos.org/plosone/s/submission-guidelines#loc-statistical-reporting>).

4. Thank you for stating the following financial disclosure:  [The funders had no role in study design, data collection and analysis, decision to publish, or preparation of the manuscript.].

At this time, please address the following queries:

a. Please clarify the sources of funding (financial or material support) for your study. List the grants or organizations that supported your study, including funding received from your institution.
b. State what role the funders took in the study. If the funders had no role in your study, please state: “The funders had no role in study design, data collection and analysis, decision to publish, or preparation of the manuscript.”
c. If any authors received a salary from any of your funders, please state which authors and which funders.
d. If you did not receive any funding for this study, please state: “The authors received no specific funding for this work.”

Please include your amended statements within your cover letter; we will change the online submission form on your behalf.

As we did not receive any funding for this study, we added the sentence “The authors received no specific funding for this work.” Page 1.

5. Please include captions for your Supporting Information files at the end of your manuscript, and update any in-text citations to match accordingly. Please see our Supporting Information guidelines for more information: [http://journals.plos.org/plosone/s/supporting-information](about:blank).

This point has been added accordingly.

## **Response to Reviewer 1:**

Thank you for your review of our paper. We have answered each of your points below.

1. The Introduction section should highlight the issues in concept drift. The contributions of the authors are not clear. They have mentioned in first contribution.

A mention to the concept drift has been added to the discussion, in the situation where the present data set was used again for further implementation. However, the present research uses a dataset and analyses gathered at one point in time, and the timing of the research does not span several years which would provoke impactful changes in the dataset. Hence, we decided to keep this in the discussion.

2. There are some paragraphs that can be merged.

In Study 1, stimuli preparation and personality measures were merged, and some of the text was added as a supporting information (S1 Text). Additional small changes were done throughout the text as to ensure that there was no overlap (e.g. in the “impressions” paragraph, we removed parts that were redundant with the conclusion).

3. We aim at extending the line of research on the acoustical bases of vocal impression in two ways: first (Study 1) by establishing a correspondence between acoustics and voice-first-impression (VFI) dimensions relative to sex, second (Study 2), by exploring the non-linear relationships between acoustical parameters and VFI by the means of machine learning models.? where is contribution and how it helps future research.

Thank you for pointing this out. Extensive modifications have been done to emphasize the contribution, the abstract has been corrected for clarity and unnecessary parts of the introduction and discussion have been removed.

Study 1 contributes by replicating a study published in 2014 (McAleer, Todorov & Belin, 2014) on the subject of first impression formation. To date, investigation of the structure of voice impression formation is still scarce, and the present results provide support for the bi-dimensional perspective, on voices.

Study 2 investigates the non-linear relationship between acoustical characteristics of voices in interaction with personality/attitudes attribution. To our best knowledge this has not been done before and literature. It successfully shows that: (1) automated classification can be used to determine perceived trustworthiness/dominance in novel voices; (2) non-linear relationships between acoustical parameters are crucial, which cannot be fully accounted for by linear regression models commonly used. On an applied perspective, it is important because it measures perceived trustworthiness and dominance in voice, which are dimensions that can be used to model synthetic speech and human-computer interaction.

4. similarly second and third contribution statement should be written in more focused way.

We think this is an excellent suggestion and emphasized those contributions in the discussion.

5. writing is good, need to check the typo errors.

Thank you for pointing this out. We proceeded to a second check and corrected the typos.

6. paper is well-formatted, plz check the formatting of the reference

Reference formatting has been checked and relevant corrections have been made.

7. I found some English mistakes please check them.

Thank you, as in point 5, we proceeded to a second check and corrected English mistakes.

8. why this specific Approach is applied for vocal impression, there is so many other statistical ways available. plz, justify it.

Thank you for your question. One of the prevalent classification method used for vocal emotion recognition in the literature is decision trees. The random forest, for instance, utilizes multiple randomly generated decision trees, which allows it to take advantage of all the benefits of decision trees, ensemble methods and bagging approaches. It extends on other bagging techniques, and exploits a random selection of the features. Another prevalent method is the Discriminant analysis. However, the SVM outperforms both discriminant analysis and logit regression (on bootstrapped samples): it provides accurate and robust classification even when inputs data are non-monotone and non-linearly separable (see Auria & Moro, 2008). In consequence, they enable to evaluate more relevant information in a convenient way. To date, it is a common method in emotional prosody classification (e.g.: Deng, Fruhholz, Zhang, Schuller, 2017), hence using it for perceived personality/attitudinal classification, which according to the emotion overgeneralization hypothesis is based on its resemblance to emotions, is a natural continuity. Hence out of consistency with literature on emotional prosody, we applied SVMs and RFs.

9. please add some more related work related to PCA..

Thank you for this suggestion. We added a very important paper published this year which supports the use of such a methodology for first impression researches:

Jones, B. C., DeBruine, L. M., Flake, J. K., Liuzza, M. T., Antfolk, J., Arinze, N. C., ... & Sirota, M. (2021). To which world regions does the valence–dominance model of social perception apply?. Nature human behaviour, 5(1), 159-169.

As for the rest of the manuscript, a large number of the sources cited here use PCA methodology. Indeed, the present experiment partially replicates McAleer, Todorov and Belin’s (2014) study on voice and first impression formation, which is described extensively throughout the paper and also uses PCA. Many other sources use PCA methodology, as common in social cognition studies, such as Oosterhof and Todorov (2008), or Sutherland et al. (2013).

10. Please cite the relevant literature but not limited to:

a) Bhattacharya, S., Maddikunta, P. K. R., Kaluri, R., Singh, S., Gadekallu, T. R., Alazab, M., & Tariq, U. (2020). A novel PCA-firefly based XGBoost classification model for intrusion detection in networks using GPU. Electronics, 9(2), 219.
b) Rehman, Z. U., Zia, M. S., Bojja, G. R., Yaqub, M., Jinchao, F., & Arshid, K. (2020). Texture based localization of a brain tumor from MR-images by using a machine learning approach. Medical hypotheses, 141, 109705.
c) Khan, H., Asghar, M. U., Asghar, M. Z., Srivastava, G., Maddikunta, P. K. R., & Gadekallu, T. R. (2021). Fake Review Classification Using Supervised Machine Learning. In Pattern Recognition. ICPR International Workshops and Challenges: Virtual Event, January 10–15, 2021, Proceedings, Part IV (pp. 269-288). Springer International Publishing.

d) Sai Ambati, L., Narukonda, K., Bojja, G. R., & Bishop, D. (2020). Factors Influencing the Adoption of Artificial Intelligence in Organizations-From an Employee's Perspective.

Thank you for suggesting this literature. Reference b has been added, however, the present paper presenting a social cognition research we estimate that it would be confusing to add GPU and network studies (a), or a pattern recognition study (c) in the literature. While they are relevant to the methodology of the research, we feel that they do not improve the understanding of the present research.

## **Response to Reviewer 2:**

Thank you for your comments. Our answers to your points are as follows.

1. Firstly, the work is of 78 pages!! which has irrelevant content as authors are deviating from the original research. It is advisable that authors should eliminate any unnecessary content under literature survey and add the data to support the literature.

Thank you for pointing this out. We agree with the reviewer’s assessment. Accordingly, in order to be more concise, a considerable reorganization and rewriting has been done.

Specifically:

- Paper is now 38 pages (without references and supporting information). As it contains two successive studies and integrates extensive statistical analyses, we think the length is justified.
- Introduction shortening (from 12 pages down to 7): we focused on the goals of the present study which are understanding the structure of impression formation, and the influence of acoustical parameters on impression formation. Thus, we removed the paragraphs on evolutionary bases (adaptive process) and integrated the vocal impressions paragraph and the personality profiles paragraph into a general introduction.
- Language has been adapted as to be more straightforward
- Unnecessary content has been moved to the supporting information (e.g. Acoustic measures in Study 1, method).
- Abstract has been rewritten (intro+conclusion)

2. Language and grammatical errors are at many places which needs to be corrected through out in the paper.

The paper has been checked and corrected for language and typos.

3. The work presented shows lack of clarity and it is not organized that any one who read the work understands it. The lack of clarity is because of unambiguous content and poor research methodology. It is advisable for authors to rephrase and redesign the methodology to support claims.

Thank you for your feedback. Multiple adjustements have been done to simplify this paper. More focused title, abstract, introduction, and results shed light on the aims of the present research. Such clarifications make the purposes of the methodology more salient: such as the use of overt judgements in Study 1, followed by automated classification in Study 2.

4. Lastly, the references are not cited properly and some references used at many places are not even in the scope of the research. Eliminate such references from the paper.

Thank you for pointing this out. Errors have been corrected. However, we could not determine which references are not in the scope or the research as noted above. Please let us know what references are problematic if they still appear in the paper after revision.

Authors are advised to cite below references in the paper

K. Chandra, G. Kapoor, R. Kohli and A. Gupta, "Improving software quality using machine learning," 2016 International Conference on Innovation and Challenges in Cyber Security (ICICCS-INBUSH), Greater Noida, India, 2016, pp. 115-118, doi: 10.1109/ICICCS.2016.7542340.


G. Arora, P. L. Pavani, R. Kohli and V. Bibhu, "Multimodal biometrics for improvised security," 2016 International Conference on Innovation and Challenges in Cyber Security (ICICCS-INBUSH), Greater Noida, India, 2016, pp. 1-5, doi: 10.1109/ICICCS.2016.7542312.

Thank you for your insight, the references cited above provide an interesting perspective to the scope of the present work. We respectfully suggest not to add these, as they are focused on cyber security and the present paper is in the scope of social cognition. We fear that adding such references would be confusing to the readers.
