# Supplementary material for: Set the tone: Trustworthy and dominant novel voices classification using explicit judgement and machine learning techniques
Source: PLoS One. 2022 Jun 29;17(6):e0267432. doi: 10.1371/journal.pone.0267432 (PMC9242519; doi:10.1371/journal.pone.0267432)
Supplement: Attachment — Submitted filename: response to reviewers.docx [file pone.0267432.s003.docx]

**[PONE-D-21-09962R1] - [EMID:87d161e3e43d220a]**

Dear reviewers,

We thank you for the time and expert attention given to our publication. We appreciate your insights and questions, which we discuss and attend to in the sections below. We are confident these adjustments improved the publication, and we hope they will meet the editor’s standards.

Please find enclosed below the responses to the reviewers comments.

Sincerely,

Cyrielle Chappuis

6. Review Comments to the Author

Please use the space provided to explain your answers to the questions above. You may also include additional comments for the author, including concerns about dual publication, research ethics, or publication ethics. (Please upload your review as an attachment if it exceeds 20,000 characters)

Reviewer #1: Good Job! Thanks for addressing the revision comments. Please make sure you have aligned the references to meet the journal requirements.

Reviewer #3: I was not involved in the first round of review. But from the response to reviewer comments, I can see that authors taken maximum care in improving the article. I have the following concerns regarding the data and experiments with machine learning:

1) You have mentioned that Forty eight native French speakers were recruited and each speaker produced 4 sentences, which should result in 48*4= 192 sentences. But you have mentioned that 160 vocal stimuli in acoustic measures section. How come this number, please check.

Thank you for bringing this to our attention. Indeed this is an oversight on our side, as we failed to mention that eight participants had been excluded from analyses due to faulty recordings. This has now been corrected.

“Forty-eight native French speakers between 18 and 35 years old (20F/20M, M = 22.47, SD =  2.61) were recruited at the University of Geneva, out of which eight participants whose recordings failed were excluded from the study »

2) Machine learning experiments: Experiments are conducted with K-fold validation. Does the authors ensured the training and testing does not consists same speaker data? If training consists of some part of the data of one speaker and testing consists of remaining data of the speaker, results may show higher accuracy due to speaker dependency. Strictly speaking, authors should conduct LOSO (leave one speaker out) cross-validation for reliability of the results as the amount is small. Please follow any standard paper for the same.

Thank you for your question and agree with the importance of this step in the classification analysis. We confirm that the SVM and RF were trained (training data) on the acoustic features of a subset of the vocal stimuli, and then were tested (testing data) to classify a new set of vocal stimuli into the possible categories. Hence, different stimuli are assessed in the training and testing.

We followed Prof. Fruhholz’s procedure for emotional voice classification as a standard (<https://www.biorxiv.org/content/10.1101/2020.05.04.076463v1.abstract>)

This step is reported page 23-24: “*The k-fold cross validation parameter was set at 5, so the training set comprises four-fifth of the data ( 50 items) while one-fifth  (13 items) is held out as testing set for each of all five experiments. Cross-validation solves the problem created by division of the dataset into a training set and a testing set by splitting the dataset into k-numbers of testing sets, subsequently fitting the model on all data, and finally computing the scores for number k of times.* »

3) Authors should present the results (performance metrics) systematically for both SVM and RF classifiers. Presently, the results are not discussed coherently in both.

We agree that the results should be presented in a coherent way in both tested classifiers, as it is paramount to the good understanding of the paper. We were very attentive to deliver the results in the most clear and well reasoned way, for both classifiers. Indeed for both classifiers, we analysed the exact same categories for both classifiers, we used the same acoustical data, and we reported accuracy for the entire classifier as well as categories. Additionnally, we identified the most important acoustical features in both classifiers when they reached their best accuracy, following a comparative approach for both. We argue that this reasoning allows to drive sufficient insight for the conclusion, and that adding more discussion to the results could make the results more opaque to the reader.

4) Authors should report the mean accuracy and standard deviation in accuracy as they are conducting experiments in K-fold validation (or LOSO). This will the show the reliability of the results.

We took this point in careful consideration, and agree that when k-fold validation is used, it is good to present mean accuracy and standard deviation to account for the variability of the results. However, in the present protocol, we fixed the random part used in the k-fold in order to have a reproducible training. In consequence, the results we report are not based on several trainings but rather on one.

5) Also authors should report the confusion matrices for both classifiers.

This point led to a careful revision of the present results. The confusion matrice for the first classifier (SVM) is reported in Figure 2, and the confusion matrice for the second classifier (RF) is reported as a table, in Table 3.

For your reference, I am giving one paper for reporting the results systematically: Glottal features for classification of phonation type from speech and neck surface accelerometer signals, Computer Speech & Language, 2021.

Otherwise, follow anyone of the standard paper which has studies on smaller amount of data.

Reviewer #4: Thanks for the authors for writing this manuscript. After related revisions, The manuscript can be accepted.
